# Mortality Outcomes for Survivors of Cancer With Food Insecurity in the US

**DOI:** 10.1001/jamahealthforum.2025.1381

**Published:** 2025-06-20

**Authors:** John C. Lin, Jiaxuan Sun, Ruiqi Yan, Lucy S. Wang, Anne Marie McCarthy, Guangyu Tong, Jaya Aysola

**Affiliations:** 1 Department of Medicine, Perelman School of Medicine, University of Pennsylvania, Philadelphia; 2 Department of Biostatistics, Epidemiology, and Informatics, Perelman School of Medicine, University of Pennsylvania, Philadelphia; 3 Penn Medicine Center for Health Equity Advancement, University of Pennsylvania Health System, Philadelphia; 4 Department of Cardiovascular Medicine, Yale School of Medicine, Yale University, New Haven, Connecticut; 5 Department of Biostatistics, Yale School of Public Health, Yale University, New Haven, Connecticut; 6 Leonard Davis Institute of Health Economics, University of Pennsylvania, Philadelphia

## Abstract

**Importance:**

Food insecurity, which is characterized by limited or uncertain access to adequate food, affects approximately 40 million individuals in the US, including many patients with cancer. Understanding the association between food insecurity and cancer mortality is crucial for improving patient outcomes and addressing health disparities.

**Objective:**

To investigate the association of food insecurity with cancer-specific and all-cause mortality among US adults with a diagnosis of cancer.

**Design, Setting, and Participants:**

This cohort study used nationally representative data from the 2011 to 2012 National Health Interview Survey that were linked to the National Death Index, with mortality follow-up through December 31, 2019. It included 5603 respondents to the National Health Interview Survey 40 years and older who reported receiving a diagnosis of cancer and responded to food security questions.

**Exposure:**

Food insecurity was measured using a validated 10-item National Center for Health Statistics food insecurity scale, categorized as food secure (0-2) or food insecure (3-10) based on US Department of Agriculture guidelines.

**Main Outcomes and Measures:**

The primary outcomes were cancer-specific mortality (*International Statistical Classification of Diseases and Related Health Problems, Tenth Revision* codes: C00-C97) and all-cause mortality. Cox proportional hazards models were used to estimate hazard ratios (HRs) for these outcomes, adjusting for age, sex, race and ethnicity, household income, US region, body mass index, smoking, alcohol use, comorbidities, and survey year.

**Results:**

Among the 5603 participants (mean [SD] age, 67.03 [12.03] years; 3298 female individuals [59%]; 320 Hispanic individuals [6%], 22 Native American individuals [0.4%], 136 non-Hispanic Asian individuals [2%], 529 non-Hispanic Black individuals [9%], 4519 non-Hispanic White individuals [81%], and 75 multiracial or multiethnic individuals [1%]), the prevalence of food insecurity was 10.3% among survivors of cancer. Food insecurity was associated with higher all-cause mortality (HR, 1.28; 95% CI, 1.07-1.53) but not cancer-specific mortality after full adjustment. Subgroup analyses indicated that food insecurity was associated with cancer and all-cause mortality for those individuals with household incomes 200% or greater than the federal poverty level (cancer-specific mortality: HR, 1.93; 95% CI, 1.18-3.15; all-cause mortality: HR, 1.89; 95% CI, 1.34-2.68) and those not receiving food assistance (cancer-specific mortality: HR, 1.42; 95% CI, 1.00-2.01; all-cause mortality: HR, 1.42; 95% CI, 1.14-1.76). However, there was no significant association between food insecurity and cancer-specific or all-cause mortality for people with incomes less than 100% of the federal poverty level and households participating in food assistance programs.

**Conclusions and Relevance:**

The results of this cohort study suggest that food insecurity is a substantial factor associated with increased mortality among survivors of cancer. Addressing food insecurity through routine screenings and connecting patients with food assistance programs may improve survival outcomes, and further research should examine the longitudinal effects of integrating routine food insecurity screenings within cancer care settings and expanding food assistance program eligibility.

## Key Points

**Question:**

How is food insecurity associated with cancer-specific and all-cause mortality in US adults with a diagnosis of cancer?

**Findings:**

In this cohort study of 5603 survivors of cancer, food insecurity was associated with higher all-cause mortality but not cancer-specific mortality after full adjustment. In subgroup analyses, food insecurity was associated with cancer and all-cause mortality among individuals with household incomes 200% or greater than the federal poverty level and those not receiving food assistance.

**Meaning:**

The study results suggest that food insecurity is associated with increased mortality in survivors of cancer, highlighting the need for routine screening and food assistance integration in cancer care to improve survival outcomes.

## Teaser

This cohort study examines the association of food insecurity with cancer-specific and all-cause mortality among US adults with a diagnosis of cancer.

## Introduction

Food insecurity, which is characterized by limited or uncertain access to adequate nutritious food, is a pervasive issue that affects approximately 40 million individuals in the US.^1^ According to the US Department of Agriculture (USDA), cancer prevalence increases as the severity of food insecurity increases, with 3.3% of food-secure adults reporting cancer compared with 5.8% of adults with very low food security.^2^ Food insecurity has been associated with higher cancer incidence and mortality in previous studies, particularly colorectal and breast cancer.^3,4,5,6^ Food insecurity due to structural and systemic issues disproportionately affects people of racial and ethnic minority groups and may play a role in racial and ethnic disparities in cancer survival.^7,8,9,10^ Research has suggested that the association between food insecurity and cancer mortality may be mediated by poor dietary choices and malnutrition.^1^ Alternatively, food insecurity may be a marker of deprivation that is associated with worse treatment adherence, suboptimal cancer screenings, and lack of access to quality care.^1^

To date, the evidence base remains unclear on the association of food insecurity with cancer mortality and the effects of age, income, and food assistance programs. Ecological studies suggest that environments with limited access to healthy food are associated with higher rates of colorectal and gastrointestinal cancer mortality.^11,12^ To our knowledge, only 2 studies (1 among a US population and the second in a Canadian population) have examined the associations between food insecurity and cancer mortality and found no significant associations.^13,14^ However, both studies included large samples of young adults, who are more likely to be food insecure and less likely to have a cancer history, and did not examine differences in cancer mortality by age, income strata, or enrollment in food assistance programs.^13,14^ Additionally, the latter study investigated cancer mortality in the general adult population, without limiting the sample to those with a history of cancer.^14^ Neither study adjusted for temporal changes despite major changes in survey methods, food insecurity, and cancer survival during their time periods^15,16^; therefore, they did not account for the major advancements in cancer care that lessened mortality.

Therefore, we investigated the association of food insecurity and cancer-specific and all-cause mortality among US adults with a diagnosis of cancer using nationally representative data from the National Health Interview Survey (NHIS) and National Death Index (NDI). We assessed the associations between food insecurity and cancer-specific and all-cause mortality, accounting for patient characteristics, behaviors, and socioeconomic indicators. We also assessed the association of food assistance programs with the association between food insecurity and mortality.

## Methods

We conducted a quantitative analysis to evaluate the association between food insecurity and mortality using 2011 to 2012 data from the NHIS that we linked with the NDI to capture mortality follow-up through December 2019. We used Cox proportional hazards models to analyze cancer-specific and all-cause mortality, adjusting for various demographic characteristics, socioeconomic characteristics, and health-related covariates. The NHIS-NDI is a publicly available, deidentified dataset; therefore, the study was deemed exempt from institutional review board review. This study follows the Strengthening the Reporting of Observational Studies in Epidemiology (STROBE) reporting guideline.

### Data Source

We used NHIS-NDI linked data for 2011 to 2012, with mortality follow-up through December 31, 2019, due to data availability. The NHIS is a cross-sectional household survey conducted annually by the National Center for Health Statistics (NCHS) that collects a range of demographic characteristics, socioeconomic characteristics, and health-related data on the civilian, noninstitutionalized population in the US.^17,18^ The 2011 to 2018 NHIS versions included questions regarding food insecurity. The NDI contains all death records for all 50 US states, Washington, DC, Puerto Rico, and the US Virgin Islands.

### Study Population

We included all individuals 40 years and older in the NHIS adult sample files that had a self-reported diagnosis of cancer and completed the food security questions.^18^ We then excluded respondents who were deemed unsuitable for NDI linkage by the NCHS, as they lacked sufficient identifying data elements. In total, 5603 respondents were included in this study.

### Food Insecurity

Our study’s independent variable was food insecurity, which was captured using a validated 10-item NCHS food insecurity scale that was scored categorically. Based on USDA guidelines, scores of 0 to 2 were considered food secure, scores of 3 to 5 were low food secure, and scores of 6 to 10 were very low food secure.^19^ We dichotomized food security into food secure (0-2) and food insecure (3-10) according to USDA classifications.^19^

### Study Outcomes

We examined outcomes associated with cancer mortality (*International Statistical Classification of Diseases and Related Health Problems, Tenth Revision *[*ICD-10*] codes: C00-C97) and all-cause mortality. Follow-up began at the date of interview through the date of death or administrative censoring (December 2019), whichever came earlier. As the NHIS-NDI database measured time in quarters, we assumed that death took place in the middle of the quarter: February, May, August, or November. Based on designations from the International Agency of Research on Cancer, we classified breast, uterine, kidney, esophageal, ovarian, liver, thyroid, stomach, pancreatic, gallbladder, and colorectal cancer as obesity-related cancers (ORCs).^20^

### Other Covariates

We examined demographic characteristics, income, health behaviors, and comorbidities. Table 1 details the complete list of covariates. We assessed age, sex, race and ethnicity, and US region per US Census designations. We obtained socioeconomic status through household income as a percentage of the federal poverty level (FPL). Health and behavior-related variables encompassed body mass index, smoking history, and alcohol consumption. We captured smoking status and alcohol consumption with validated measures with the following categories: never, former, and current. The survey captured alcohol consumption through the question, “In your entire life, have you had at least 12 drinks of any type of alcoholic beverage?”^18^ We derived comorbidities using the Charlson Comorbidity Index, which was adapted to the NHIS based on previous research.^21,22,23^ We recorded receipt of food assistance based on whether respondents’ households participated in the Supplemental Nutrition Assistance Program (SNAP) or the Special Supplemental Nutrition Program for Women, Infants, and Children (WIC).^18^ We excluded respondents with missing data for food insecurity, cancer survivorship, and mortality. For all other categorical covariates, missing values were included in a missing category (eTable 1 in Supplement 1).^24^

**Table 1.** Characteristics of Survivors of Cancer by Food Security Status

Characteristics	No. (%)		*P* value
	Food secure	Food insecure	
Total, No.	5024	579	NA
Age, y			
40-50	490 (10)	122 (21)	<.001
51-60	879 (18)	183 (32)	
61-70	1474 (29)	164 (28)	
≥71	2181 (43)	110 (19)	
Sex			
Female	2920 (58)	378 (65)	.001
Male	2103 (42)	201 (35)	
Race and ethnicity			
Hispanic	244 (5)	76 (13)	<.001
Native American	14 (0.3)	8 (1)	
Non-Hispanic Asian	120 (2)	16 (3)	
Non-Hispanic Black	414 (8)	115 (20)	
Non-Hispanic White	4170 (83)	349 (60)	
Multiracial or multiethnic	60 (1)	15 (3)	
Household income, % FPL			
<100	381 (9)	218 (41)	<.001
100-199	776 (18)	192 (36)	
200-399	812 (19)	70 (13)	
≥400	2298 (54)	55 (10)	
US Region			
Northeast	837 (17)	89 (15)	.04
Midwest	1196 (24)	119 (21)	
South	1772 (35)	238 (41)	
West	1219 (24)	133 (23)	
BMI, mean (SD)	30.24 (14.46)	31.56 (12.89)	.08
Smoking			
Never	2444 (49)	215 (38)	<.001
Former	1959 (39)	144 (25)	
Current	574 (12)	213 (37)	
Alcohol use			
Never	963 (20)	112 (20)	<.001
Former	1188 (24)	216 (38)	
Current	2784 (56)	236 (42)	
Charlson Comorbidity Index score			
0-3	1506 (30)	94 (16)	<.001
4-5	2076 (41)	208 (36)	
6-14	1442 (29)	277 (48)	
Cancer type^a^			
Breast	995 (20)	100 (17)	.15
Lung	132 (3)	20 (3)	.25
Prostate	655 (13)	53 (9)	.01
Colorectal	356 (7)	45 (8)	.54
Uterus	201 (4)	48 (8)	<.001
Other^b^	2128 (42)	315 (54)	<.001

Abbreviations: BMI, body mass index (calculated as weight in kilograms divided by height in meters squared); FPL, federal poverty level; NA, not applicable.

^a^ Participants may have had multiple types of cancer.

^b^ Other includes cancers of the bladder, blood, bone, brain, cervix, esophagus, gallbladder, kidney, larynx, leukemia, liver, lymphoma, melanoma, mouth/tongue/lip, ovary, pancreas, skin, soft tissue, stomach, testis, pharynx, thyroid, and other.

### Statistical Analysis

We compared respondents by food security status using descriptive statistics, Pearson χ^2^ tests for categorical variables, and 1-way analysis of variance for continuous variables. We also tested interactions of food insecurity with age and income and evaluated the association between food assistance and income using χ^2^ tests. We fitted Cox proportional hazards models to evaluate the association between food insecurity and all-cause and cancer-specific mortality for patients with cancer.^25^ We used Schoenfeld residuals to check for deviation from the proportional hazards assumption. We used cumulative incidence graphs to depict the estimated cumulative probabilities of mortality over time as stratified by different levels of food security. To evaluate the association of specific covariates with the association of food insecurity and mortality, we developed 4 sequential models that adjusted for specific covariates. Model 1 was adjusted for age and survey year. Model 2 additionally included demographic variables, including sex, race and ethnicity, and US region. Model 3 built on model 2 by adding income. Finally, model 4 encompassed behavioral health variables (body mass index, smoking history, alcohol consumption) and degree of comorbid conditions (Charlson Comorbidity Index) in addition to model 3.

Additionally, in subgroup analyses using model 4, we estimated all-cause and cancer-specific mortality risk among food assistance programs enrollees compared with nonenrollees. We further examined the associations between the independent variable and dependent variable within each income strata, ORC group, and age strata as defined previously given the association of household income with food insecurity and access to cancer care, as well as age strata with survival outcomes.^21^ Subgroup analysis by cancer type was not possible due to limited statistical power (eTable 2 in Supplement 1). Given the potential confounding associations of smoking and income, we then examined cancer-specific and all-cause mortality in those who never smoked with household incomes greater than 200% of the FPL. We also conducted sensitivity analysis using the 3-tiered definition of food insecurity as defined previously. All analyses were performed in Stata, version 18.0 (StataCorp), and R, version 4.2.3 (R Core Team). Statistical significance was set at α = .05.

## Results

In total, 5603 people were included in this study. The prevalence of food insecurity among survivors of cancer and people without cancer diagnoses were 10.3% and 13.1%, respectively. Survivors of cancer with food insecurity were more likely to be younger (122 [21%] were age 40-50 years vs 490 [10%] without food insecurity), female (378 [65%] vs 2920 [58%]), Black (115 [20%] vs 414 [8%]) or Hispanic (76 [13%] vs 244 [5%]), and have household incomes less than 100% of the FPL (218 [41%] vs 381 [9%]; Table 1).

### Association Between Food Security and Mortality

Hazard ratios are shown in Table 2; ; Kaplan-Meier survival plots are shown in the Figure. We observed 546 deaths due to cancer, with a median follow-up of 7.8 years (IQR, 7.3-8.3 years, range, 0-8.9 years), including 65 deaths associated with food insecurity and 481 with food security. Age-adjusted risk of cancer mortality was higher for people with food insecurity (HR, 1.52; 95% CI, 1.17-1.99) compared with those with food security and remained higher after adjusting for demographic characteristics and geography. Adjusting for income attenuated cancer mortality risk. Accounting for behavioral covariates and comorbidities did not significantly alter cancer risk compared with previous models. Interaction with food security was not statistically significant for age or income.

**Table 2.** Association of Food Insecurity and Mortality in Cox Proportional Hazards Models^a^

Food security status	Hazard ratio (95% CI)			
	Model 1	Model 2	Model 3	Model 4
**Cancer-specific mortality**				
Food security (n = 5024)	1 [Reference]	1 [Reference]	1 [Reference]	1 [Reference]
Food insecurity (n = 579)	1.52 (1.17-1.99)^b^	1.47 (1.12-1.93)^b^	1.31 (0.98-1.75)	1.23 (0.92-1.64)
**All-cause mortality**				
Food security (n = 5024)	1 [Reference]	1 [Reference]	1 [Reference]	1 [Reference]
Food insecurity (n = 579)	1.66 (1.41-1.95)^c^	1.69 (1.43-2.00)^c^	1.36 (1.14-1.63)^c^	1.28 (1.07-1.53)^b^

^a^ Four sequential models were developed to evaluate the association between food insecurity and mortality. Model 1 adjusted for age and survey year. Model 2 included these factors along with demographic variables, such as sex, race and ethnicity, and US region. Model 3 further added household income. Model 4 additionally incorporated behavioral health factors (body mass index, smoking history, alcohol consumption) and the Charlson Comorbidity Index.

^b^ *P* < .01.

^c^ *P* < .001.

**Figure.**
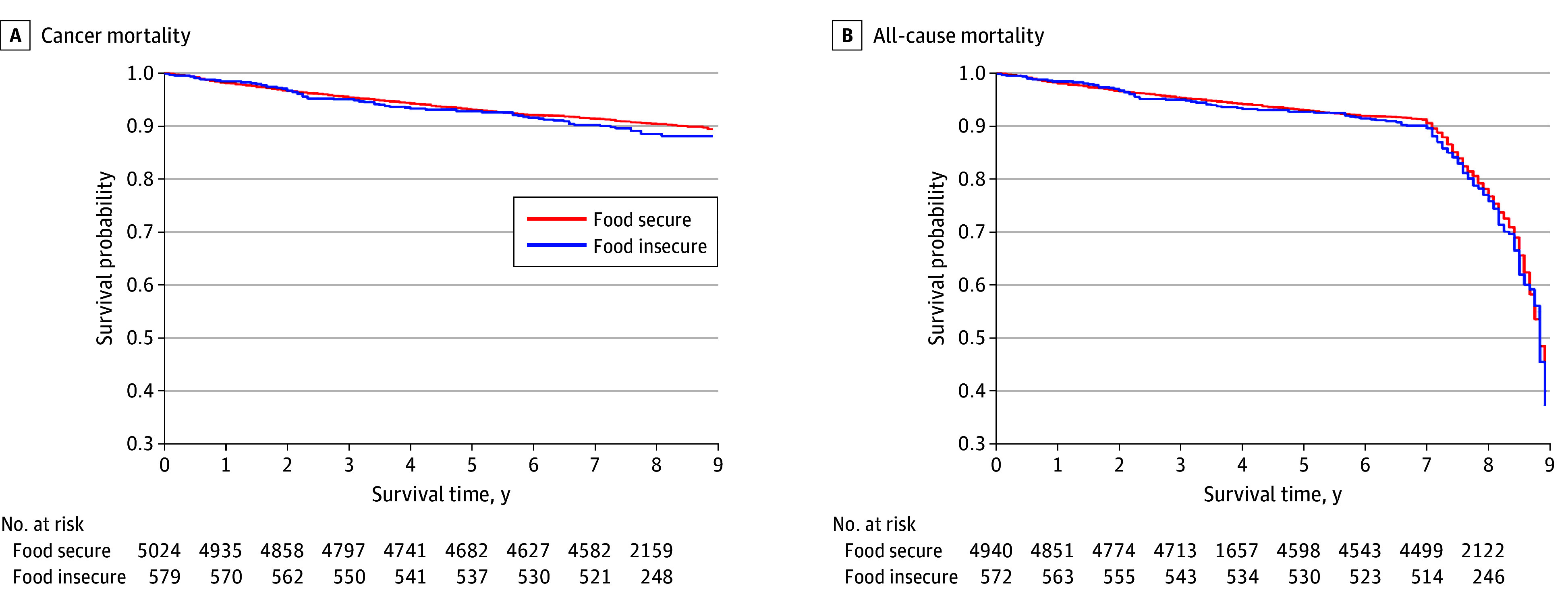
Kaplan-Meier Survival Estimates for Survivors of Cancer With Food Insecurity

In total, 1556 deaths of all causes were observed, with a median follow-up of 7.6 years (IQR, 7.0-8.3 years; range, 0-8.9 years), including 169 deaths associated with food insecurity and 1387 with food security. Age-adjusted risk of all-cause mortality was higher for people with food insecurity (HR, 1.66; 95% CI, 1.41-1.95) compared with those with food security, which persisted after adjusting for demographic characteristics (HR, 1.69; 95% CI, 1.43-2.00), socioeconomic characteristics (HR, 1.36; 95% CI, 1.14-1.63), and behavioral covariates and comorbidities (HR, 1.28; 95% CI, 1.07-1.53). Interaction with food security was not statistically significant for age or income.

In households participating in food assistance programs (Table 3), food insecurity was not associated with cancer-specific mortality (HR, 0.75; 95% CI, 0.44-1.29) nor all-cause mortality (HR, 0.87; 95% CI, 0.63-1.21). However, for households not receiving food assistance, food insecurity was associated with cancer-specific (HR, 1.42; 95% CI, 1.00-2.01) and all-cause mortality (HR, 1.42; 95% CI, 1.14-1.76). For people with household incomes 200% or greater than the FPL (Table 4), food insecurity was associated with cancer-specific (HR, 1.93; 95% CI, 1.18-3.15) and all-cause mortality (HR, 1.89; 95% CI, 1.34-2.68). However, this association was not significant for people with incomes less than 100% of the FPL for cancer-specific (HR, 0.62; 95% CI, 0.33-1.19) and all-cause mortality (HR, 0.78; 95% CI, 0.55-1.11). Receiving food assistance was associated with household income; most people with incomes less than 100% of the FPL received food assistance (320 of 598 [54%]), but not people with household incomes between 100% to 200% of the FPL (161 of 968 [17%]) or greater than 200% of the FPL (72 of 3235 [2%]) (*P* < .001).

**Table 3.** Adjusted Associations Between Food Insecurity and Mortality by Food Assistance Program Enrollees and Nonenrollees^a^^,^^b^

Food security status	Assistance, hazard ratio (95% CI)			
	Food insecurity (n = 604)	Total No.	No food insecurity (n = 4999)	Total No.
**Cancer mortality**				
Food security	1 [Reference]	369	1 [Reference]	4655
Food insecurity	0.75 (0.44-1.29)	235	1.42 (1.00-2.01)^c^	344
**All-cause mortality**				
Food security	1 [Reference]	369	1 [Reference]	4655
Food insecurity	0.87 (0.63-1.21)	235	1.52 (1.22-1.89)^d^	344

^a^ Food assistance programs include the Supplemental Nutrition Assistance Program or the Special Supplemental Nutrition Program for Women, Infants, and Children.

^b^ Table 3 analyses adjust for age, survey year, sex, race and ethnicity, US region, household income, body mass index, smoking history, alcohol consumption, and the Charlson Comorbidity Index.

^c^ *P* < .05.

^d^ *P* < .001.

**Table 4.** Adjusted Associations Between Food Insecurity and Mortality by Household Income^a^

Food security status	<100% of the FPL (n = 599)		100% to 199% of the FPL (n = 968)		≥200% of the FPL (n = 3235)		
	HR (9% CI)	Total No.	HR (95% CI)	Total No.	HR (95% CI)	Total No.	
**Cancer mortality**							
Food security	1 [Reference]	1 [Reference]	1 [Reference]	1 [Reference]	1 [Reference]		3110
Food insecurity	0.62 (0.33-1.19)	218	1.39 (0.88-2.22)	192	1.93 (1.18-3.15)^b^		125
**All-cause mortality**							
Food security	1 [Reference]	381	1 [Reference]	776	1 [Reference]		3110
Food insecurity	0.78 (0.55-1.11)	218	1.36 (1.02-1.81)^c^	192	2.04 (1.44-2.89)^d^		125

Abbreviations: FPL, federal poverty level; HR, hazard ratio.

^a^ Table 4 analyses adjust for age, survey year, sex, race and ethnicity, US region, body mass index, smoking history, alcohol consumption, and the Charlson Comorbidity Index.

^b^ *P* < .01.

^c^ *P* < .05.

^d^ *P* < .001.

When subgrouped by ORC status (eTable 6 in Supplement 1), food insecurity was associated with cancer mortality in model 1 (adjusting for age) among people with ORCs (HR, 1.49; 95% CI, 1.01-2.20) and non-ORCs (HR, 1.52; 95% CI, 1.06-2.18). However, these associations were not significant in model 4 after adjusting for all covariates. For ORCs, food insecurity was associated with all-cause mortality in model 1 (HR, 1.72; 95% CI, 1.34-2.20) and model 4 (HR, 1.32; 95% CI, 1.01-1.74); this association was only significant for model 1 (HR, 1.56; 95% CI, 1.26-1.94) for non-ORCs.

We conducted further subgroup analyses by age as well as smoking status and household income. When subgrouped by age (eTable 3 in Supplement 1), food insecurity was associated with cancer-specific mortality (HR, 1.58; 95% CI, 1.01-2.47) and all-cause mortality (HR, 1.56; 95% CI, 1.14-2.14) for people aged 61 to 70 years. For those who never smoked with household incomes greater than 200% of the FPL (eTable 4 in Supplement 1), food insecurity was associated with cancer-specific (HR, 3.39; 95% CI, 1.46-7.86) and all-cause mortality (HR, 2.13; 95% CI, 1.09-4.17).

We also completed sensitivity analysis with a 3-tiered food insecurity scale (eTable 5 in Supplement 1). When adjusting for age alone, low food security was associated with cancer-specific (HR, 1.63; 95% CI, 1.19-2.25) and all-cause mortality (HR, 1.59; 95% CI, 1.30-1.95), and very low food security was associated with cancer-specific mortality (HR, 1.72; 95% CI, 1.34-2.20). These results became nonsignificant when adjusting for all covariates.

## Discussion

In this cohort study, we analyzed the association between food insecurity and mortality for adult survivors of cancer in the US using NHIS and NDI data. After adjusting for demographic characteristics, income, health and behaviors, and comorbidities, we found that food insecurity was associated with all-cause mortality, but not cancer-specific mortality. Accounting for income appeared to render insignificant cancer-specific mortality risk. Our analyses also revealed the associations between food insecurity and mortality risk varied by income strata and participation in food assistance programs. We found that food insecurity was associated with all-cause mortality for those from middle-income households (100%-199% of the FPL) and with cancer-specific and all-cause mortality for those from higher-income households (greater than or equal to 200% of the FPL) and those who did not receive food assistance. Additionally, we found that food insecurity was significantly associated with all-cause mortality for ORCs compared with non-ORCs in adjusted models that accounted for obesity and comorbidities, although the differences in HRs were modest.

Evidence suggests that food insecurity contributes to cancer mortality through myriad pathways. Food-insecure households that prioritize energy-dense and nutrient-poor foods may contribute to cancer recurrence, in association with mortality.^1,7,8,9,26^ Food insecurity may also be associated with reduced consumption of essential nutrients, such as vegetables, fruits, and dairy, exacerbating the risk of obesity and its associated complications, such as cancer and mortality.^27^ Additionally, dietary exposures to carcinogens (which are common in less expensive foods) may contribute to cancer pathogenesis.^28^ Moreover, the financial strain of cancer care may compound food insecurity and compromise treatment adherence, as patients may struggle to afford their treatment costs and food.^1^

Experiencing food insecurity can be a vicious cycle that can worsen disease outcomes, drive additional health care expenditures, and strain financial resources, thereby worsening food insecurity.^29^ People with food insecurity often confront difficult trade-offs between food and other necessities, like medicine or medical care, particularly in the absence of adequate insurance coverage.^30,31^ Moreover, the finding of the association of food insecurity with all-cause mortality highlighted its association with conditions beyond cancer.^14^ The USDA reports that food insecurity compared with income has a greater association with many chronic illness.^2^ Research has shown that food insecurity is associated with obesity, diabetes, hypertension, coronary heart disease, hepatitis, stroke, cancer, asthma, arthritis, chronic obstructive pulmonary disease, and kidney disease.^2,32^ Prior studies also found associations between food insecurity and worse cardiovascular mortality among US adults.^33,34^ Food insecurity may be particularly concerning for cardiovascular mortality in survivors of cancer, who may be susceptible to higher rates of cardiovascular toxic effects due to prior radiotherapy and chemotherapy.^35,36^

We found that food insecurity was associated with cancer-specific and all-cause mortality for people not receiving food assistance and for those with household incomes greater than or equal to 200% of the FPL, who mostly do not qualify for SNAP or WIC benefits.^37^ These findings highlight that households with higher incomes that do not qualify for food assistance programs experience food insecurity that is significantly associated with cancer and all-cause mortality. It also suggested that food assistance programs may mitigate the association of food insecurity with cancer mortality, aligning with prior research on the benefits of such programs.^38^ Moreover, our study showed that not only do survivors of cancer with household incomes greater than the cutoff for SNAP and WIC benefits experience food insecurity, but also that their food insecurity is associated with cancer-specific and all-cause mortality.

We also found that food insecurity was associated with cancer-specific and all-cause mortality for people aged 61-70 years. One potential reason for this may stem from individuals at this age transitioning into retirement, during which they begin receiving Social Security benefits.^39^ The receipt of Social Security benefits, while often insufficient to fully support financial needs, can disqualify older adults from accessing SNAP.^39,40^ Prior studies have revealed that older adults are the least likely to use SNAP compared with other age groups in the US.^39,40^

### Implications for Policy and Practice

Nearly half of US adults who report food insecurity do not participate in food assistance programs.^41^ Major barriers include a lack of knowledge about programs, especially about personal eligibility, as well as psychological, social, and legal pressures.^42,43,44^ Therefore, there is a need to (1) screen for food insecurity and enroll those who are eligible for assistance programs and (2) expand income eligibility criteria for food assistance programs to meet the growing food insecurity needs among the US adult population.

### Practice-Based Screening

The association between food insecurity and poor health outcomes has prompted advocacy for routine food insecurity screenings in clinical settings, facilitating early intervention to mitigate its detrimental associations with patient health and alleviate its burden on the US health care system.^1^ Medical organizations, such as the American Academy of Pediatrics, American Diabetes Association, and American Heart Association, have recommended food insecurity screenings.^33,45,46^ Furthermore, the American Society for Clinical Oncology has recommended systematic data collection through expanded screenings for health-related social needs and social determinants of health.^47^ However, to our knowledge, there have not been formal recommendations by the American Cancer Society, Society for Surgical Oncology, American Society for Radiation Oncology, or American Society of Hematology. Given the prevalent nature of food insecurity among survivors of cancer and its association with health outcomes, societies should consider position statements that call for expanded food insecurity screening efforts in clinical oncology.^1^

### Practice-Based Interventions and Advocacy

Physicians can play a role in addressing food insecurity for patients with cancer and survivors. Evidence from a 2022 randomized clinical trial suggested that food vouchers and access to food pantries in safety-net cancer clinics were associated with improved treatment completion, food security, depression, and quality of life among patients with cancer and food insecurity.^48^ Health care professionals can also connect patients with community resources, such as food banks, meal delivery services, or government food assistance programs.^33,45,46^ Collaborating with registered dietitians or nutritionists can help produce tailored dietary plans that accommodate patients’ nutritional needs and financial constraints.^33,45,46^ Patient education on budget-friendly meal preparation and strategies for maximizing nutrition on a limited budget can empower individuals to make healthier food choices despite financial challenges.^33,45,46^ Furthermore, clinician and community advocacy for policy changes to address systemic issues contributing to food insecurity, such as income inequality and inadequate social safety nets, can create lasting improvements in patients’ access to food and overall well-being.^33,45,46^

### Policy Changes

State and federal governments should also consider expanding income eligibility to federal food assistance programs, considering compelling evidence linking food insecurity with adverse health outcomes, including mortality for survivors of cancer. The expansion of food benefits, as exemplified by initiatives in California, Massachusetts, and North Carolina, may substantially alleviate the burden of food insecurity and improve health outcomes while lowering health care costs.^33^ These benefits can address nutritional deficiencies, mitigate the risk of chronic diseases, and enhance treatment adherence and quality of life for individuals undergoing cancer treatment.^49^ Moreover, these initiatives align with broader public health goals of reducing health disparities and promoting health equity.^49^

### Additional Considerations

Using cancer mortality as an outcome may obscure differences across cancer types, and there may be variability in specifying cancer as the cause of death. Using cancer mortality as an adjunct to all-cause mortality helps to avoid potential biases based on data classification.^50^ Moreover, although the NDI is limited by its reliance on death certificates, it has also been shown to improve the accuracy of survival outcomes for cancer registries.^51,52^ Additionally, different cancer types may have different physiological effects on the human body, and the lack of statistical power limited our ability to conduct subgroup analyses by cancer type. However, food insecurity may be akin to other factors that prior research has identified to be associated with cancer outcomes irrespective of cancer types, such as financial hardship, being uninsured, or experiencing homelessness.^53,54,55^ Future studies should continue to investigate how food insecurity affects deaths of specific cancers and other causes of mortality.

### Limitations

This study had several limitations. First, we used self-reported data on cancer diagnoses, although self-reported cancer diagnoses have been validated in previous survey research in the US.^56^ Second, food security status was analyzed based on a single point within the NHIS dataset, which may not fully capture fluctuations in food insecurity status over time. Third, the NHIS dataset also lacks data on cancer stage, remission, or treatment. Although we controlled for many potential confounders, there remains a possibility of residual confounding that could have affected the observed associations.

## Conclusions

The findings of this cohort study emphasized the importance of addressing food security in cancer care to improve overall survival outcomes. As frontline health care clinicians, physicians have a responsibility to recognize and address food insecurity among patients. Expanding the eligibility for food assistance programs, coupled with integrating routine food security screenings into clinical practice and connecting individuals with appropriate resources, may help mitigate the associations of food insecurity with mortality for survivors of cancer.

## Notes

Supplement 1. eTable 1. Proportion of Missing Data Across Covariates in the Analysis eTable 2. Mortality and Food Insecurity Status Across Selected Cancer Types eTable 3. Association of Food Insecurity and Mortality By Age eTable 4. Association of Food Insecurity and Mortality in Cox Proportional Hazards Models for Never-Smokers with Household Incomes over 200% FPL eTable 5. Association of Food Insecurity and Mortality in Cox Proportional Hazards Models Using Three-Tiered Food Insecurity Scale eTable 6. Association of Food Insecurity and Mortality in Cox Proportional Hazards Models by Cancer Type

Supplement 2. Data sharing statement
