# Supplementary material for: Mortality Outcomes for Survivors of Cancer With Food Insecurity in the US
Source: JAMA Health Forum. 2025 Jun 20;6(6):e251381. doi: 10.1001/jamahealthforum.2025.1381 (PMC12181791; doi:10.1001/jamahealthforum.2025.1381)
Supplement: Supplement 1. — eTable 1. Proportion of Missing Data Across Covariates in the Analysis eTable 2. Mortality and Food Insecurity Status Across Selected Cancer Types eTable 3. Association of Food Insecurity and Mortality By Age eTable 4. Association of Food Insecurity and Mortality in Cox Proportional Hazards Models for Never-Smokers with Household Incomes over 200% FPL eTable 5. Association of Food Insecurity and Mortality in Cox Proportional Hazards Models Using Three-Tiered Food Insecurity Scale eTable 6. Association of Food Insecurity and Mortality in Cox Proportional Hazards Models by Cancer Type [file jamahealthforum-e251381-s001.pdf]

Supplemental Online Content

Lin JC, Sun J, Yan R, et al. Mortality outcomes for survivors of cancer with food insecurity in the US. *JAMA Health Forum*. 2025;6(6): e251381. doi:10.1001/jamahealthforum.2025.1381

eTable 1. Proportion of Missing Data Across Covariates in the Analysis
eTable 2. Mortality and Food Insecurity Status Across Selected Cancer Types
eTable 3. Association of Food Insecurity and Mortality By Age
eTable 4. Association of Food Insecurity and Mortality in Cox Proportional Hazards Models for Never-Smokers with Household Incomes over 200% FPL
eTable 5. Association of Food Insecurity and Mortality in Cox Proportional Hazards Models Using Three-Tiered Food Insecurity Scale
eTable 6. Association of Food Insecurity and Mortality in Cox Proportional Hazards Models by Cancer Type

This supplemental material has been provided by the authors to give readers additional information about their work.

© 2025 Lin JC et al. *JAMA Health Forum*.

eTable 1: Proportion of Missing Data Across Covariates in the Analysis

Characteristics (N=5,603)	Missing, n	Missing, %
Age	0	0.00%
Sex	1	0.02%
Race/Ethnicity	2	0.04%
Household Income	801	14.30%
US Region	0	0.00%
BMI	0	0.00%
Smoking	54	0.96%
Alcohol Usage	104	1.86%
Charlson Comorbidity Index	0	0.00%
Cancer Type	0	0.00%

© 2025 Lin JC et al. *JAMA Health Forum*.

eTable 2: Mortality and Food Insecurity Status Across Selected Cancer Types

Food security status	Sample Size	Deaths	Cancer Deaths
**Breast Cancer**			
Food security	995	258	98
Food insecurity	100	27	8
**Lung Cancer**			
Food security	132	75	52
Food insecurity	20	10	6
**Prostate Cancer**			
Food security	655	234	65
Food insecurity	53	17	9
**Colorectal Cancer**			
Food security	356	135	39
Food insecurity	45	14	7
**Melanoma**			
Food security	374	100	28
Food insecurity	32	12	6
**Kidney Cancer**			
Food security	103	36	14
Food insecurity	15	3	2

© 2025 Lin JC et al. *JAMA Health Forum*.

eTable 3: Association of Food Insecurity and Mortality By Age

**Cancer Mortality, Hazard Ratio (95% CI)**				
**Food security status**	**40-50 years**	**51-60 years**	**61-70 years**	**71+ years**
Food security	1 [reference]	1 [reference]	1 [reference]	1 [reference]
Food insecurity	1.15 (0.33-3.95)	1.03 (0.56-1.90)	**1.58 (1.01-2.47)***	1.16 (0.69-1.94)
**All-Cause Mortality, Hazard Ratio (95% CI)**				
**Food security status**	**40-50 years**	**51-60 years**	**61-70 years**	**71+ years**
Food security	1 [reference]	1 [reference]	1 [reference]	1 [reference]
Food insecurity	1.19 (0.50-2.83)	1.14 (0.77-1.71)	**1.59 (1.16-2.18)****	1.28 (0.97-1.70)

*p<0.05; **p<0.01; ***p<0.001. CI, confidence interval.

© 2025 Lin JC et al. *JAMA Health Forum*.

eTable 4: Association of Food Insecurity and Mortality in Cox Proportional Hazards Models for Never-Smokers with Household Incomes over 200% FPL

**Cancer Mortality, Hazard Ratio (95% CI)**		
**Food security status**	**Model 1 (age)**	**Model 4 (+ behaviors, comorbidities)**
Food security (n=1546)	1 [reference]	1 [reference]
Food insecurity (n=45)	**3.65 (1.67-7.98)****	**3.39 (1.46-7.86)****
**All-cause Mortality, Hazard Ratio (95% CI)**		
**Food security status**	**Model 1 (age)**	**Model 4 (+ behaviors, comorbidities)**
Food security (n=1546)	1 [reference]	1 [reference]
Food insecurity (n=45)	**2.28 (1.24-4.20)****	**2.13 (1.09-4.17)***

© 2025 Lin JC et al. *JAMA Health Forum*.

eTable 5: Association of Food Insecurity and Mortality in Cox Proportional Hazards Models Using Three-Tiered Food Insecurity Scale

**Cancer Mortality, Hazard Ratio (95% CI)**		
**Food security status**	**Model 1 (age)**	**Model 4 (+ behaviors, comorbidities)**
Food security (n=5024)	1 [reference]	1 [reference]
Low food security (n=315)	**1.63 (1.19-2.25)****	1.36 (0.97-1.91)
Very low food security (n=264)	1.36 (0.90-2.06)	1.08 (0.70-1.68)
**All-cause Mortality, Hazard Ratio (95% CI)**		
**Food security status**	**Model 1 (age)**	**Model 4 (+ behaviors, comorbidities)**
Food security (n=5024)	1 [reference]	1 [reference]
Low food security (n=315)	**1.59 (1.30-1.95)*****	1.24 (0.999-1.54)
Very low food security (n=264)	**1.72 (1.34-2.20)*****	1.27 (0.97-1.65)

© 2025 Lin JC et al. *JAMA Health Forum*.

eTable 6. Association of Food Insecurity and Mortality in Cox Proportional Hazards Models by Cancer Type[a]

**Type**	**HR (95% CI)**			
	**Food security status**	**Deaths, No.**	**Model 1 (age)**	**Model 4 (+ demographic characteristics, income, behaviors, comorbidities)**
**Cancer mortality**				
ORCs[b]	Food security (n = 1883)	188	1 [Reference]	1 [Reference]
	Food insecurity (n = 247)	31	1.49 (1.01-2.20)	1.23 (0.80-1.90)
Non-ORCs[b]	Food security (n = 3141)	293	1 [Reference]	1 [Reference]
	Food insecurity (n = 332)	34	1.52 (1.06-2.18)	1.21 (0.81-1.80)
**All-cause mortality**				
ORCs	Food security (n = 1883)	529	1 [Reference]	1 [Reference]
	Food insecurity (n = 247)	76	1.72 (1.34-2.20)	1.32 (1.01-1.74)
Non-ORCs	Food security (n = 3141)	858	1 [Reference]	1 [Reference]
	Food insecurity (n = 332)	93	1.56 (1.26-1.94)	1.14 (0.89-1.45)

Abbreviations: HR, hazard ratio; ORC, obesity-related cancer.
[a]Table 5 analyses adjust for age, survey year, sex, race and ethnicity, US region, household income, body mass index, smoking history, alcohol consumption, and the Charlson Comorbidity Index.
[b]Participants were classified as having ORCs if they reported having breast, uterine, kidney, esophageal, ovarian, liver, thyroid, stomach, pancreatic, gallbladder, and colorectal cancer. Non-ORCs included people who only had other types of cancers.

© 2025 Lin JC et al. *JAMA Health Forum*.
