# Supplementary material for: Mortality Outcomes for Survivors of Cancer With Food Insecurity in the US
Source: JAMA Health Forum. 2025 Jun 20;6(6):e251381. doi: 10.1001/jamahealthforum.2025.1381 (PMC12181791; doi:10.1001/jamahealthforum.2025.1381)
Supplement: Supplement 2. — Data sharing statement [file jamahealthforum-e251381-s002.pdf]

Data Sharing Statement

Lin. Mortality Outcomes for Survivors of Cancer With Food Insecurity in the US. *JAMA Health Forum*. Published June 20, 2025. doi:10.1001/jamahealthforum.2025.1381

Data
Data available: Yes
Data types: Deidentified participant data, Data dictionary
How to access data: All data and documentation are available on websites published by the National Center for Health Statistics, Centers for Disease Control and Prevention (https://www.cdc.gov/nchs/nhis/data-questionnaires-documentation.htm).
When available: With publication

Supporting Documents
Document types: Other (please specify)
Additional Information: All data and documentation are available on websites published by the National Center for Health Statistics, Centers for Disease Control and Prevention (https://www.cdc.gov/nchs/nhis/data-questionnaires-documentation.htm).
How to access documents: All data and documentation are available on websites published by the National Center for Health Statistics, Centers for Disease Control and Prevention (https://www.cdc.gov/nchs/nhis/data-questionnaires-documentation.htm).
When available: With publication

Additional Information
Who can access the data: All data and documentation are available on websites published by the National Center for Health Statistics, Centers for Disease Control and Prevention (https://www.cdc.gov/nchs/nhis/data-questionnaires-documentation.htm).
Types of analyses: For any purpose.
Mechanisms of data availability: Without investigator support.
